# (Es)ketamine in functional neurological disorder: a systematic review

**DOI:** 10.3389/fpsyt.2026.1872389

**Published:** 2026-07-20

**Authors:** Bruce Tamilson, Michael Chitolie, Jan Coebergh, Niruj Agrawal, Norman Poole, Matt Butler, Richard Kanaan, Timothy Nicholson

**Affiliations:** 1 Neuropsychiatry Service, South West London & St George’s Mental Health NHS Trust, St George’s Hospital, London, United Kingdom; 2 Department of Neuroscience, St George’s University of London, London, United Kingdom; 3 Division of Psychiatry, University College of London, London, United Kingdom; 4 Department of Liaison Psychiatry, Kingston Hospital, London, United Kingdom; 5 Atkinson Morley Regional Neurosciences Centre, St George’s University Hospital, London, United Kingdom; 6 Institute of Psychiatry, Psychology, and Neuroscience, King’s College London, London, United Kingdom; 7 Department of Psychiatry, University of Melbourne, Melbourne, VIC, Australia

**Keywords:** Esketamine, ketamine, functional neurological disorder (FND), functional/dissociative seizures, conversion disorder

## Abstract

**Background and aim:**

Functional neurological disorder (FND) is a common and disabling condition for which effective treatments remain limited. Clinical outcomes are often variable, particularly in chronic and comorbid presentations. Ketamine and esketamine are increasingly used across a range of neuropsychiatric conditions. This review aimed to synthesise the existing evidence regarding their use in FND.

**Methods:**

A systematic review of PubMed, PsycINFO and Google Scholar was conducted up to December 2025. Studies reporting clinical outcomes following (Es)ketamine administration in functional neurological symptoms were included. Owing to substantial clinical and methodological heterogeneity, findings were synthesised narratively.

**Results:**

Thirteen studies were identified, predominantly comprising case reports and small case series. Across the included studies, some patients were reported to experience improvement in functional motor symptoms, functional dissociative seizures, and other functional presentations following ketamine or esketamine administration. Interpretation of these findings is limited by the uncontrolled nature of the evidence base, heterogeneity of diagnoses and outcomes, and the potential influence of publication and expectancy bias. Adverse effects were variably reported and were generally described as transient in supervised clinical settings.

**Conclusion:**

The current evidence base is limited to predominantly uncontrolled and highly heterogeneous studies and is insufficient to establish the efficacy of (Es)ketamine for FND. Although improvements in functional symptoms have been reported across several FND presentations, it remains unclear whether these reflect direct effects on functional symptoms, indirect benefits arising from psychiatric improvement, or other non-specific therapeutic factors. While no firm conclusions regarding efficacy can be drawn, the existing data suggest that further investigation is justified, and prospective controlled trials are needed to clarify the potential role of (Es)ketamine in FND.

## Introduction

Functional neurological disorder (FND) is a common and disabling condition characterised by motor, sensory, cognitive, and seizure symptoms that arise from altered nervous system functioning rather than structural neurological disease (1). Contemporary models increasingly conceptualise FND as a disorder of nervous system functioning involving alterations in functional brain network processing, in which alterations in attention, symptom expectations, perception, emotion, self-agency, and motor control may contribute to symptom generation and maintenance, although the precise mechanisms remain incompletely understood (2).

Despite advances in diagnostic understanding and increasing recognition of FND as a potentially reversible condition, many patients experience persistent symptoms, impaired quality of life, and substantial functional disability (3). Current treatment approaches emphasise multidisciplinary care incorporating psychoeducation, allied health therapies (physiotherapy, Occupational therapy, Speech and Language therapy) and psychological interventions. Nevertheless, treatment outcomes remain variable, particularly among individuals with chronic illness and psychiatric comorbidity (4).

There is a historical precedent for the use of dissociative pharmacological compounds in the treatment of FND. Evidence of the use of nitrous oxide, an NMDAR receptor modulator, in the treatment of symptoms resembling FND can be seen as far back as 1799 (5). Early abreaction paradigms, often utilising sodium amytal or barbiturates, often alongside suggestion therapy, has some suggestive evidence base (6, 7). More recent evidence from a case series described the successful use of propofol, at dissociative sub-anaesthetic doses, alongside physiotherapy in the treatment of motor functional neurological disorder (8).

Ketamine and its S-enantiomer, esketamine (hereafter collectively referred to as ‘(Es)ketamine’), are increasingly used and studied in neuropsychiatric practice, particularly for treatment-resistant depression (9, 10). As clinical use of (Es)ketamine has expanded, reports have emerged describing improvement in functional neurological symptoms among individuals receiving these agents either for FND itself or for comorbid psychiatric conditions. Given the substantial clinical heterogeneity of FND, findings from one symptom domain cannot necessarily be generalised to others, highlighting the need for a systematic synthesis of the available evidence.

(Es)ketamine is associated with transient alterations in perception, embodiment, and subjective experience during administration, commonly described as dissociative phenomena (11). This may be particularly relevant in FND, where dissociative symptoms and altered bodily experiences occur in a subset of patients. However, the clinical significance of these experiences remains unclear. At present, there is insufficient evidence to determine whether such experiences influence treatment response, are clinically neutral, or may have adverse effects in susceptible individuals (12).

To date, no systematic review has specifically examined the use of (Es)ketamine in FND. Given the limited and heterogeneous nature of the available evidence, this systematic review aimed to identify, synthesise, and critically evaluate the published literature describing (Es)ketamine use in individuals with FND. Specifically, we sought to characterise the clinical contexts in which these agents have been used, summarise reported clinical outcomes and adverse effects, examine methodological strengths and limitations within the existing evidence base, and identify priorities for future research. In the absence of controlled studies, the objective of this review was not to determine treatment efficacy, but rather to provide a systematic and critical synthesis of the currently available clinical observations.

## Methods

### Design and reporting

A systematic review with narrative synthesis was conducted to identify and synthesise the published evidence describing the use of (Es)ketamine in individuals with FND and related functional neuropsychiatric presentations. Given the anticipated clinical and methodological heterogeneity of the literature, quantitative synthesis and meta-analysis were not planned. The review was not prospectively registered.

### Eligibility criteria

Studies were eligible if they involved adolescents or adults with FND or historically related diagnostic constructs characterised by clearly described functional neurological symptoms. Eligible diagnoses included contemporary FND presentations as well as older terminology such as conversion disorder and selected FND-adjacent presentations used in historical literature. Eligible interventions were ketamine or esketamine administered by any route. Studies were included when (Es)ketamine targeted either functional symptoms directly or comorbid psychiatric disorders, provided that outcomes relating to functional neurological symptoms were reported. Clinical trials, observational studies, case series, case reports, and cross-sectional studies were eligible. Conference abstracts, dissertations, non-peer-reviewed reports, and non-English publications were excluded.

### Diagnostic boundary and case-definition strategy

Both the terminology and conceptualisation surrounding FND have evolved substantially over time, with considerable overlap between FND and historically used constructs including conversion disorder, psychogenic disorders, dissociative presentations, and selected somatoform conditions. To maximise sensitivity and avoid excluding potentially relevant historical literature, studies describing related functional presentations were considered eligible when they involved neurological symptoms that would reasonably align with contemporary FND concepts.

During data extraction, historical diagnostic labels and case definitions were mapped onto contemporary FND classifications to ensure consistent study classification. Purely psychiatric dissociative disorders without functional neurological manifestations were excluded. Given the heterogeneity of included presentations, findings were interpreted cautiously and were not assumed to generalise uniformly across motor, seizure, sensory, dissociative, and somatic phenotypes.

### Information sources and search strategy

Electronic searches were conducted in Google Scholar and PubMed between 1 January and 20 January 2026 and covered literature from database inception to 31 December 2025. Following peer-review suggestion, a supplementary retrospective search of PsycINFO was undertaken using the same search strategy, using the same end-date restriction, to assess whether any additional eligible studies had been missed.

Because FND terminology has changed considerably over time, a broad free-text search strategy was adopted. Terms relating to ketamine exposure (“ketamine”, “esketamine”, and “NMDA antagonist”) were paired individually with terms reflecting both contemporary and historical FND nomenclature, including: “functional neurological disorder”; “conversion disorder”; “functional symptoms”; “psychogenic”; “non-epileptic seizures”; “PNES”; “functional movement disorder”; and “dissociative”. Searches were conducted separately for each paired combination. The complete search strategy and number of records retrieved for each search are provided in **Supplementary Table 1**.

Google Scholar was included because of its broad indexing of older literature, case reports, and publications that may not be consistently captured within PubMed. Across all paired searches, Google Scholar returned 5,962 total retrievals. Google Scholar records were screened in order of relevance ranking. For search combinations yielding more than 1,000 results, screening was limited to the first 1,000 relevance-ranked records.

Citations identified through Google Scholar searches were saved to the Google Scholar “My Library” function throughout the search process. Because Google Scholar automatically identifies records already saved to a user’s library, this approach facilitated management of overlapping search results generated by multiple search term combinations and reduced repeated assessment of duplicate records. Following completion of all searches, unique Google Scholar citations were exported for formal screening.

PubMed searches were conducted using identical paired search terms. Retrieved records were imported into EndNote and compared against the Google Scholar citation library to identify potentially unique records requiring screening. A retrospective PsycINFO search was subsequently performed using the same paired search terms while excluding PubMed-indexed records to minimise duplication. Finally, reference lists of all included studies and relevant review articles were manually screened to identify any additional eligible publications.

### Study selection

Unique Google Scholar records were exported and imported into EndNote (Clarivate Analytics). PubMed and PsycINFO records were subsequently imported and compared against the existing library. Following duplicate removal, records underwent title and abstract screening, followed by full-text assessment of potentially eligible studies. Although pragmatic, the use of a single reviewer for study screening and selection may have increased the risk of selection bias. Several measures were undertaken to minimise this risk, including the use of broad search terms encompassing historical and contemporary FND terminology, supplementary database searches, manual screening of reference lists, and conservative inclusion of studies for full-text assessment when eligibility was unclear. Nevertheless, the possibility of missed studies cannot be excluded and is acknowledged as a limitation. Studies meeting eligibility criteria proceeded to data extraction and quality appraisal.

Title and abstract screening, full-text assessment, and study selection were performed by a single reviewer (BT), whereas data extraction and quality appraisal were conducted independently by two reviewers (BT and MC), with discrepancies resolved through discussion and consensus.

### Data extraction

Data extraction was independently performed by BT and MC using a standardised extraction form. Extracted variables included study characteristics, year and country of publication, study design, sample size, participant demographics, functional symptom subtype, ketamine or esketamine formulation, dose, route of administration, treatment protocol, psychiatric comorbidity, outcome measures, therapeutic effects, duration of follow-up, and reported adverse events. Extracted data were cross-checked between reviewers prior to synthesis.

### Quality assessment

Methodological quality was independently assessed by BT and MC using Joanna Briggs Institute (JBI) critical appraisal tools appropriate to each study design. Disagreements were resolved through discussion and consensus.

### Data synthesis

Given the substantial clinical and methodological heterogeneity of included studies, including variation in diagnostic terminology, symptom phenotype, intervention protocols, and outcome assessment, findings were synthesised narratively and grouped according to clinical presentation and evidence type.

## Results

A total of 5,962 records were identified through Google Scholar searches. Following relevance-based screening and removal of duplicate records across search combinations, 2,524 unique Google Scholar citations were retained for formal screening. PubMed searches yielded 501 records, of which 30 represented potentially unique citations after comparison with the Google Scholar library. A retrospective PsycINFO search identified 80 records; however, no additional eligible studies were identified. Manual screening of reference lists from included studies and relevant review articles did not identify any further eligible publications.

**Figure 1:**
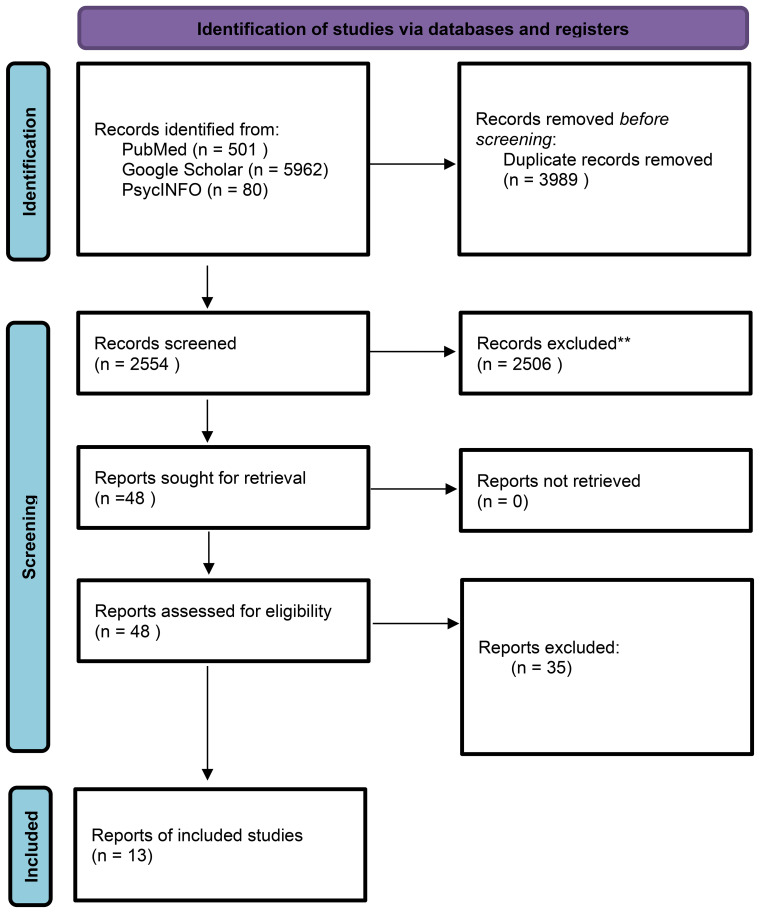
PRISMA Flow diagram adapted from: Page et al., 2021 (13).

Thirteen studies met eligibility criteria and were included in the final qualitative synthesis and quality appraisal. The study selection process is summarised in **Figure 1**.

### Quality assessment of included studies

The methodological quality of included studies was assessed using JBI critical appraisal tools matched to study design (14). Quality of studies ranged from poor to high. Most studies were single-case reports, evaluated with the JBI Case Report Checklist. Recent reports (2021–2025) showed moderate to high quality (6.5–8/8), with clear reporting of patient characteristics, diagnostics, interventions, and outcomes. Case series were appraised using the JBI Case Series Checklist. A recent ketamine case series by Tham et al. demonstrated high quality (10/10), while older publications scored poorly (2–3/10). One cross-sectional online survey, assessed with the JBI Prevalence Checklist, showed moderate quality (7/9), limited by non-probability sampling and self-reported diagnoses.

Overall, the evidence base was characterised by a predominance of uncontrolled observational designs, small sample sizes, limited use of validated outcome measures, and short or inconsistently reported follow-up periods, resulting in a generally low level of certainty regarding treatment effects.

### Study characteristics and evidence overview

The evidence base was limited and consisted predominantly of uncontrolled observational reports. The 13 included studies comprised two historical narcoanalytic reports, nine case reports, one small case series, and one cross-sectional survey examining self-directed psychoactive substance use. Clinical presentations included functional dissociative seizures (FDS), functional motor symptoms, dissociative presentations, and other FND-adjacent functional syndromes. The review included studies comprising 70 individuals with FND or FND-adjacent presentations, including 63 clinical cases/cohort participants and 7 survey respondents reporting ketamine use (**Table 1**).

**Table 1:** Characteristics of included studies.

Author	Design	Evidence category	Setting	Functional symptoms	Comorbid conditions	Primary target	Psychotherapy/ rehabilitation	Outcome assessment*	Follow up duration in weeks	JBI score	Key limitations
Khorramzadeh & Lotfy 1973 Iran (15)	Open-label clinical series Total (n = 100) Functional conditions (n = 30)	Historical narcoanalytic/ abreactive report	Psychiatric Inpatient	FDS/PNES (n = 13); Conversion reaction (n = 11), dissociative reaction (n = 6)	Not available	Conversion disorder/FND Adjacent conditions	Psychodynamic psychotherapy	Clinician-observed symptom improvement	26	2/10	No control group; outdated diagnostic framework and outcomes; unclear standardized follow-up
Golechha et al., 1986 India (16)	Controlled clinical study (n = 45) Ketamine treated (n = 30)	Historical controlled narcoanalytic study	Outpatient psychiatry	Conversion disorder or FND adjacent conditions (n = 22)	Not available	Conversion disorder/FND Adjacent conditions	Part of broader psychiatric treatment	Clinician-observed abreaction and symptom response	3	3/10	Pre-DSM framework; short follow-up; No measurement of improvement of FND symptoms
Raviteja & Harihar, 2018 India (17)	Case report 15M	Contemporary case report	Inpatient psychiatry	Dissociative amnesia	Acute stress	FND-adjacent	Psychotherapy	Recovery of memory/function and clinical follow-up	1	7/8	Paediatric case; non-generalizable
Vendrell-Serres et al., 2021 Spain (18)	Case report 49M	Contemporary TRD-esketamine case report	Outpatient psychiatry	Functional Movement Disorder	Treatment-resistant depression	TRD	Ongoing psychiatric care and antidepressant treatment	Clinical assessment of functional weakness + depression scales	21	7/8	No control; unclear durability; mood-mediated effects
Moccia et al., 2021 Italy (19)	Case report 57F	Contemporary TRD-esketamine case report	Outpatient psychiatry	Functional movement disorder	TRD, complicated grief	TRD	Concurrent psychiatric management and antidepressant	Psychogenic Movement Disorders Rating Scale (PMDRS) + depression scales	26	8/8	Single case; improvement parallels mood response
Tham et al., 2022 Canada (20)	Case series Total (n = 10) FND (n = 2)	Case series	Inpatient psychiatry	Functional Dissociative Seizures/PNES (n- 2)	Severe TRD, anxiety, medical comorbidity	TRD	Multidisciplinary psychiatric care	Depression rating scales (MADRS, QIDS-SR16)	12	10/10	Not designed for FND outcomes; small sample
Butler et al., 2023 UK & Int (21).	Online survey FND (n = 1162) Ketamine use (n = 7)	Survey/self-directed use	Online survey	FND	Not available	Not Applicable	Not applicable	Self-reported survey responses	Not applicable	7/9	Self-report; non-medical use; recall and expectancy bias, online sample, recruitment bias (patient charity)
Argento et al., 2023 USA (22)	Case report 51F	Contemporary ketamine-assisted therapy case report	Outpatient psychiatry	Functional Dissociative Seizures/PNES	MDD, PTSD, fibromyalgia	FSD + MDD + PTSD	Psychotherapy	Seizure frequency, functional ability, quality of life	23	8/8	Single case; concurrent psychotropics; psychotherapy confound; expectancy effects
Wang et al., 2024 China (23)	Case report 51F	FND-adjacent case report	Inpatient psychiatry	FND-adjacent somatic symptom presentation	Depression, anxiety	SSD/depression (FND-adjacent)	Psychiatric treatment	Somatic symptom improvement + psychiatric scales	3	8/8	SSD ≠ FND;
Hashmi et al., 2025 USA (24)	Case report 45F	Contemporary TRD-esketamine case report	Outpatient TRD clinic	Functional Dissociative Seizures/PNES	TRD, PTSD, anxiety	TRD	Routine psychiatric treatment	PNES frequency and clinical status	39	8/8	No objective seizure counts; case-level evidence
Cameron-Burr et al., 2025 USA (25)	Case report 23F	FND-adjacent case report	Emergency department	Paradoxical vocal cord motion (PVCM)	Anxiety, depression, GORD, post-COVID	PVCM/FND-adjacent symptoms	Prior and ongoing psychiatric and medical care	Clinical symptom resolution and discharge from ED	Nil	8/8	Acute-only; non-DSM FND; no follow-up
Kimata et al., 2025 USA (26)	Case report 28M	Emergency case report	Emergency department	Acute functional motor paralysis	Bipolar I disorder, anxiety, chronic pain	Acute FND	None reported	Clinical symptom resolution in ED	Nil	8/8	Short observation
Rijal et al., 2025 Nepal (27)	Case report 53F	Contemporary case report	Inpatient psychiatry	Dissociative disorder with somatic symptoms	Depression, anxiety traits	FND-adjacent	Psychotherapy	Clinical improvement + BDI/HAM-A	39	6.5/8	Single case concurrent psychotropic adjustment

FDS, Functional Dissociative Seizures; PNES, Psychogenic Non-Epileptic Seizures; TRD, Treatment-Resistant Depression; PTSD, Post-Traumatic Stress Disorder; MDD, Major Depressive Disorder; SSD, Somatic Symptom Disorder; PMDRS, Psychogenic Movement Disorders Rating Scale; MADRS, Montgomery–Asberg Depression Rating Scale; QIDS-SR16, Quick Inventory of Depressive Symptomatology–Self-Report (16-item); BDI, Beck Depression Inventory; HAM-A, Hamilton Anxiety Rating Scale; GORD, Gastro-Oesophageal Reflux Disease; PVCM, Paradoxical Vocal Cord Motion; ED, Emergency Department; JBI, Joanna Briggs Institute; M, male; F, female.

Treatment intent varied substantially across the included studies. Historical narcoanalytic reports and abreaction-based case reports generally administered ketamine with the explicit aim of facilitating recovery from functional or dissociative symptoms, including conversion disorder, psychogenic non-epileptic seizures, dissociative amnesia, and dissociative presentations with somatic symptoms. More recent emergency department reports similarly used sub-dissociative ketamine to target acute functional symptom exacerbations, including functional motor paralysis and paradoxical vocal cord motion. In contrast, several contemporary studies prescribed (Es)ketamine primarily for TRD, often in the presence of comorbid functional neurological symptoms. In these reports, improvements in functional symptoms were observed as secondary outcomes occurring alongside improvements in mood and other psychiatric symptoms.

Concurrent therapeutic interventions were common. Historical studies used ketamine within narcoanalytic or psychotherapeutic frameworks, often incorporating suggestion and facilitated emotional processing. Several contemporary reports involved ongoing psychiatric care, antidepressant treatment, (Es)ketamine-assisted psychotherapy, or multidisciplinary mental health management. In some cases, participants were also receiving concurrent medical, rehabilitative or medical interventions. Consequently, the independent contribution of (Es)ketamine to reported clinical improvement was frequently difficult to determine.

Outcome assessment methods varied considerably across studies. Most relied on clinician-observed symptom change, patient self-report, seizure frequency, functional recovery, or emergency department disposition. Psychiatric symptom scales were used in several studies targeting depression; however, only one study employed a validated FND-specific outcome measure. Follow-up duration ranged from acute emergency department observation to approximately one year, although longer-term outcomes were inconsistently reported.

### Early abreactive and narcoanalytic approaches

Abreaction refers to the therapeutic process of re-experiencing and releasing emotions linked to previously repressed or dissociated traumatic memories, traditionally thought to reduce symptoms through emotional discharge and integration (28). Narcoanalysis (e.g., the amobarbital interview) involves administering sedative medication to lower inhibition and increase verbal responsiveness, allowing patients to access and articulate otherwise inaccessible thoughts, memories, or conflicts (29). It has been used in the past for diagnostic clarification and to facilitate abreaction in conditions such as conversion and dissociative disorders.

Two early studies explored ketamine as an abreactive or narcoanalytic agent in functional and dissociative disorders. Khorramzadeh and Lotfy reported outcomes from a cohort of 100 patients. Of these, 30 had functional symptoms, including dissociative reactions, conversion reactions, and functional dissociative seizures, and were treated with ketamine-assisted abreaction (15). At six-month follow-up, the majority of patients demonstrated clinical improvement, with sustained benefit reported at one year in most participants (88 out of total 100 patients). Only a small minority continued to experience significant symptoms at long-term follow-up. The authors also described individual cases in which ketamine-facilitated psychotherapy was associated with rapid symptom resolution and early hospital discharge. However, the study employed an uncontrolled open-label design, used historical diagnostic constructs, and relied on clinician-reported outcomes without standardised assessment measures. Consequently, the durability and specificity of the reported treatment effects are difficult to determine.

Similarly, Golechha et al. conducted a narcoanalysis study using ketamine (n = 30) alongside other pharmacological agents (control group n = 15) in patients with functional seizures, psychogenic aphonia, functional pain syndromes, and other somatoform presentations (16). Presenting symptom accentuation and heightened emotional arousal were commonly (60% of the cases in both intervention and control groups) observed. However, these phenomena were regarded as expected effects of narcoanalysis. Formal assessment of subsequent functional symptom improvement was not reported. As a result, the study provides evidence that ketamine can facilitate abreactive experiences but offers little direct evidence regarding improvement in functional neurological symptoms, as symptom outcomes were not systematically measured or reported.

### Acute presentation of FND symptoms

More recent literature (Kimata et al., 2025) has described rapid improvement of FND symptoms following sub-dissociative ketamine administration in an acute medical context. In this study, a case of a young adult admitted to an emergency department with acute functional motor paralysis, triggered by a fall, was reported, in whom intravenous sub-dissociative ketamine resulted in prompt symptom resolution and facilitated discharge from hospital (26). The authors emphasised ketamine’s potential adjunctive role in managing acute FND presentation, particularly where symptom burden delays medical discharge. However, interpretation of this observation is limited by the single-case design, absence of a control condition, and short observation period. Acute functional neurological symptoms can fluctuate considerably, and spontaneous improvement may occur, particularly following diagnostic clarification, reassurance, changes in clinical context, or resolution of acute physiological and psychological stressors (30). Furthermore, outcome assessment was mostly based on immediate clinical observation rather than validated FND-specific measures, and no longer-term follow-up was reported.

### Functional/dissociative seizures

Three case reports described improvement in FDS following (Es)ketamine treatment. Argento et al., 2023 reported a patient with refractory daily seizures, comorbid PTSD, MDD and fibromyalgia who underwent a structured ketamine-assisted therapy programme (22). Following an initial three-week induction and extended intermittent ketamine treatment combined with integrative psychotherapy, the patient demonstrated substantial reductions in seizure frequency and severity, alongside meaningful improvements in mood and functional capacity. However, interpretation is complicated by the concurrent delivery of psychotherapy, the prolonged treatment period, multiple psychiatric comorbidities, and the absence of objective seizure monitoring or validated FND outcome measures.

Tham et al., 2022 reported, within a 10-patient case series of subcutaneous racemic ketamine for treatment-resistant depression, that two patients with comorbid conversion disorder/FND and daily-to-weekly seizure-like attacks experienced essentially complete remission of seizure-like episodes, sustained at three-month follow-up (20). Notably, ketamine was prescribed primarily to treat depression rather than FDS, and outcomes were assessed using depression rating scales rather than FND-specific measures. Given the very small sample size and the concurrent improvement in affective symptoms, it remains unclear whether seizure reduction reflected a direct effect on functional symptoms, secondary benefits arising from psychiatric improvement, or other non-specific treatment factors.

Hashmi et al., 2025 described a single patient with TRD and PNES/FDS who reportedly experienced cessation of seizure episodes after initiating intranasal esketamine, alongside marked improvement in depressive symptoms, with HAM-D decreasing from 23 to 7 (24). Although the temporal association is notable, interpretation is limited by the uncontrolled single-case design, lack of objective seizure quantification, and the inability to disentangle potential effects of psychiatric symptom improvement, expectancy effects, ongoing psychiatric care, or spontaneous symptom fluctuation.

### Functional movement disorder

Two case reports documented significant improvement or remission of chronic FMD following treatment with intranasal esketamine, particularly in individuals with comorbid TRD. Moccia et al., 2021 reported a 57-year-old woman with chronic FMD dating from 1993, characterised by trunk torsions, genuflections and severely impaired gait. Following adjunctive intranasal esketamine, initially 56 mg twice weekly, then 84 mg weekly, complete remission of motor symptoms was observed within two months, with progressive improvement in depressive symptoms and full affective remission by month four, sustained at six months (19). The study was strengthened by specialist neurological assessment and the use of the Psychogenic Movement Disorders Rating Scale (PMDRS); however, conclusions remain limited by the uncontrolled single-case design and concurrent psychiatric treatment.

Comparable findings were reported by Vendrell-Serres et al., 2021, who described a 49-year-old man with FND characterized by sensory and motor paralysis of the left upper limb and comorbid TRD. Following initiation of esketamine nasal spray, improvement in motor and sensory symptoms was observed from the first treatment session, with eventual restoration of full limb function and sustained benefit during follow-up (18). However, the patient was simultaneously receiving ongoing psychiatric care, antidepressant treatment, and cognitive behavioural therapy targeting both depressive and functional symptoms, making it difficult to isolate the specific contribution of esketamine to the observed clinical improvement.

### FND-adjacent presentations

Four reports described improvement following (Es)ketamine administration in presentations adjacent to, but not fully representative of, contemporary FND classifications. Interpretation of these findings therefore requires particular caution.

Wang et al., 2024 reported rapid improvement in refractory somatic symptom disorder (functional postural and motor symptoms) with comorbid depression in a 51-year-old woman with a five-year history of feeling that her body was “pulled to the right” after neck surgery. After a second intravenous esketamine infusion at 0.4 mg/kg, depressive, anxiety, and somatic symptoms improved markedly, with residual symptoms limited to the face and neck; functional recovery was described as substantial (23). However, the case was diagnosed as SSD, not FND, so its diagnostic relationship to contemporary FND is uncertain. Interpretation is also limited by the single-case design, extensive prior antidepressant, psychological, acupuncture, rTMS, scopolamine, and stellate ganglion block interventions, and uncertainty about whether somatic improvement reflected a direct effect on physical symptoms, secondary effects of mood improvement, or their interaction.

The Raviteja and Harihar 2018 case report described a 15-year-old boy with eight days of dissociative amnesia, including loss of previously fluent languages, who recovered memories and language ability during a single intramuscular ketamine abreaction session at 1 mg/kg, with total remission reported at one-week and two-month follow-up (17). However, the authors framed ketamine as an abreactant used to facilitate recall of traumatic memories, not as pharmacotherapy for dissociative symptoms per se. Recovery also occurred alongside supportive psychotherapy, discussion of psychosocial stressors with parents, family reassurance, removal from acute stressors, and the known potential for acute dissociative/conversion symptoms to improve spontaneously, so attribution specifically to ketamine is limited.

Similarly, Rijal et al., 2025 described a single case of suspected dissociative disorder and depressive symptoms in which ketamine-assisted abreaction was followed by psychotherapeutic exploration of psychosocial stressors, coping-skills interventions, and ongoing antidepressant treatment. Clinical improvement was observed and reportedly maintained during follow-up; however, the multimodal nature of the intervention and the concurrent use of desvenlafaxine preclude attribution of benefit specifically to ketamine (27).

A similar pattern was described by Cameron-Burr et al., 2025, who reported successful emergency department treatment of paradoxical vocal cord motion (PVCM), a functional disorder, using low-dose intravenous ketamine (25). Symptom resolution occurred within 30 minutes of ketamine initiation, allowing safe discharge and avoidance of escalation to intensive care, suggesting a potential role for ketamine in selected acute functional presentations. While clinically noteworthy, PVCM occupies a heterogeneous position at the interface of functional, psychiatric, respiratory, and laryngeal disorders. The reported patient had multiple potential contributing factors, including anxiety, depression, gastro-oesophageal reflux disease, tracheobronchomalacia, and post-COVID sequelae. Standard interventions, including benzodiazepines and reflux-directed treatment, had also been administered prior to ketamine. Consequently, although the temporal association between ketamine administration and symptom resolution was striking, the relative contribution of ketamine compared with concurrent treatments, spontaneous symptom resolution, or other non-specific factors remains uncertain.

### Patient-reported experiences and self-directed use

Survey-based data were limited but informative. Butler et al., 2023 conducted a large international online survey exploring self-management strategies and patient attitudes toward managing FND (21). A small subset of respondents (n =7) reported illicit ketamine use for symptom management, rating perceived effectiveness relatively highly (Median perceived effectiveness 72/100). Most individuals reported minimal physical or psychological adverse effects. The authors emphasised cautious interpretation but highlighted ketamine-assisted therapy as a potential future research direction, particularly for patients with motor FND and comorbid depression.

Across heterogeneous study designs, (Es)ketamine were consistently associated with rapid symptom improvement in a subset of patients with FND, including motor symptoms, functional dissociative seizures, and other functional presentations. Reported benefits often emerged quickly and, in several cases, were sustained over weeks to months. Improvement frequently occurred alongside reductions in depressive and trauma-related symptoms. Nevertheless, the evidence base remains limited by uncontrolled study designs, high risk of expectancy and placebo effects, reliance on subjective outcome assessment, inconsistent diagnostic confirmation, and the absence of validated FND-specific outcome measures.

### (Es)ketamine administration regimens across studies and reported adverse effects across studies

Across the included studies, ketamine and esketamine were administered using a wide range of formulations, routes, dosing strategies, and treatment settings (**Table 2**). Racemic ketamine was delivered intravenously, intramuscularly, or subcutaneously, whereas esketamine was administered intranasally or intravenously. Dosing ranged from sub-dissociative doses used in acute emergency presentations to dissociative-range dosing employed in ketamine-assisted psychotherapy and abreaction procedures. Treatment schedules varied substantially, ranging from single administrations to repeated protocols extending over several months. Emergency department reports utilised single low-dose intravenous ketamine infusions, while historical narcoanalytic studies and contemporary abreaction reports typically employed single treatment sessions designed to facilitate emotional processing or symptom exploration. In contrast, studies involving treatment-resistant depression generally used repeated ketamine administrations or standard intranasal esketamine protocols over weeks to months.

**Table 2:** Administration of (Es)ketamine and reported adverse effects across studies.

Author	Relevant functional conditions	Drug	Route	Dose	Regime	Outcome	Adverse effects
Khorramzadeh & Lotfy 1973 Iran (15)	Conversion disorder and FND adjacent conditions	Racemic ketamine	IV	0.2–0.3, 0.4–0.6, 0.7–1.0 mg/kg (abreaction paradigm)	Single administration	Symptom relief described across diagnostic groups. All of the subjects were seen 6 months after the injection. Only 9 patients were not doing well at this time.	Minor complications only: apprehension (2), nausea (3), vomiting (2). Overall described as “very minimal.”
Golechha et al., 1986 India (16)	Conversion disorder and FND adjacent conditions	Ketamine	IM	0.5-1.5mg/Kg	Single administration	Accentuation of presenting symptoms in 60% of the cases.	Transient effects: floating sensation, diplopia, nystagmus, limb numbness. Nausea/giddiness lasting 12–16 h in 5 cases (mostly at 1.5 mg/kg). No emergence delirium. No long-term sequelae.
Raviteja & Harihar. 2018 India (17)	Dissociative amnesia	Racemic ketamine	IM	1 mg/kg	Single administration	Full recovery of memory and language	Transient drowsiness/cataplexy lasting 15 minutes
Vendrell-Serres et al., 2021 Spain (18)	FMD	Esketamine	IN	56–84 mg	Standard TRD protocol*	Reported Complete remission of motor symptoms and depression	Mild dissociative symptoms, resolving within 2 hours; no other adverse effects reported.
Moccia et al., 2021 Italy (19)	FMD	Esketamine	IN	56–84 mg	Weekly dosing over 4 months	Reported Full resolution of abnormal movements	No adverse effects explicitly reported in the case narrative.
Tham et al., 2022 Canada (20)	FDS	Racemic ketamine	SC	0.5–1 mg/kg	Twice weekly; up to 6 sessions	Reported complete remission of seizures and Improvement in depressive symptoms	Mild, transient dissociation (CADSS mean <1); transient BP elevation (8–10 mmHg systolic); no severe AEs; no cognitive deterioration; no treatment discontinuation.
Butler et al., 2023 UK & Int (21).	FND	Ketamine	NA	Not reported	Not reported	Median perceived effectiveness ≈72/100	Ketamine: mostly minimal physical/psychological effects when used.
Argento et al., 2023 USA (22)	FDS	Racemic ketamine	SL/IN	Individualised dissociative dosing	3-week intensive phase + 20-week maintenance	Marked reduction in seizure frequency and severity	No significant somatic or psychological side effects reported; post-session fatigue only.
Wang et al., 2024 China (23)	FND-adjacent somatic symptom presentation	Esketamine	IV	0.2 → 0.4 → 0.5 mg/kg	Three 40-min infusions over 13 days	Rapid improvement of functional and mood symptoms	Mild dizziness. It was transient and well tolerated.
Hashmi et al., 2025 USA (24)	FDS	Esketamine	IN	56–84 mg	Standard TRD protocol*	Resolution of FDS episodes; antidepressant response	Patient reported no side effects throughout treatment.
Cameron-Burr et al., 2025 USA (25)	PVCM	Racemic ketamine	IV	0.15 mg/kg over 15 min (sub-dissociative)	Single administration	Rapid symptom resolution	No adverse effects reported in case; patient returned to baseline mental status.
Kimata et al., 2025 USA (26)	Acute functional motor paralysis	Racemic ketamine	IV	0.15 mg/kg (sub-dissociative)	Single administration	Rapid reversal of motor symptoms	No adverse effects observed. Discussion notes laryngospasm as a rare theoretical risk, not observed.
Rijal et al., 2025 Nepal (27)	Dissociative disorder with somatic symptoms	Racemic ketamine	IV	0.2 mg/kg (intermittent bolus)	Single administration	Rapid symptom relief and emotional processing	Brief confusion (10–15 min) post-session; headache, relieved with paracetamol.

*-Standard Treatment Resistant Depression (TRD) esketamine protocol: It is administered intranasally alongside an oral antidepressant, using a structured regimen. Treatment begins with twice-weekly dosing (56–84 mg) for four weeks, followed by weekly dosing for a further four weeks, and then maintenance every one to two weeks (31).

Across the included studies, (Es)ketamine were generally reported to be well tolerated, with adverse effects predominantly transient and mild (**Table 2**). In medically supervised settings, the most consistently described adverse effects were short-lived dissociative or perceptual symptoms, typically resolving within the observation period, usually within one to two hours. Similar findings were reported across a range of treatment contexts, including intranasal esketamine for treatment-resistant depression, subcutaneous ketamine, intravenous sub-dissociative administration in emergency settings, and ketamine-assisted psychotherapy. Historical narcoanalytic studies likewise reported predominantly transient adverse effects, although nausea, vomiting, dizziness, diplopia, and subjective perceptual disturbances appeared more common at higher intramuscular doses.

Autonomic effects were infrequently reported and were generally mild, consisting primarily of transient increases in blood pressure or heart rate that resolved without intervention. Gastrointestinal symptoms, including nausea, dizziness, headache, and transient confusion, were reported sporadically and did not result in treatment discontinuation. No study reported persistent cognitive impairment, psychosis, serious cardiorespiratory complications, or treatment-related hospitalisation. Similarly, the survey study examining self-directed psychoactive substance use in individuals with FND found that most respondents reported no or minimal adverse physical or psychological effects associated with ketamine use.

However, interpretation of the apparent safety profile requires caution. Most included studies were single case reports or small case series with limited follow-up, and adverse-event monitoring was rarely a primary study objective. Reporting of adverse effects was frequently brief, non-standardised, and based on clinician observation or patient self-report rather than systematic assessment. Furthermore, many reports focused primarily on clinical improvement and provided limited detail regarding adverse-event ascertainment, duration, or severity. Long-term safety outcomes were largely unavailable, and the absence of reported adverse effects should not be interpreted as evidence that such effects did not occur. Importantly, individuals experiencing clinically significant adverse events may be less likely to be represented within the published case-report literature, introducing the potential for publication and reporting bias. Consequently, while the available literature suggests that (Es)ketamine were generally tolerated in the reported cases, the current evidence base is insufficient to draw firm conclusions regarding safety in broader FND populations.

## Discussion

This systematic review identified a small and highly heterogeneous body of literature describing the use of (Es)ketamine in individuals with FND and related functional presentations. Reported improvements were observed across a range of symptom domains, including functional dissociative seizures, functional motor symptoms, dissociative presentations, and selected FND-adjacent conditions. In several reports, improvements occurred rapidly and, where follow-up was available, were maintained for weeks or months. Reported adverse effects were generally mild and transient. However, the available evidence consisted entirely of historical narcoanalytic studies, single case reports, one small case series, and self-reported survey data. No randomised controlled trials, controlled observational studies, or prospective cohorts specifically designed to evaluate ketamine-based interventions in FND were identified. Consequently, the current evidence base does not permit firm conclusions regarding efficacy.

### Interpreting reported improvement

A central challenge in interpreting the current literature is determining what mechanism, if any, accounts for the reported improvement in functional symptoms following (Es)ketamine administration. Given the absence of controlled studies and the frequent presence of psychiatric comorbidity, concurrent psychotherapy, and multidisciplinary treatment, multiple competing explanations remain plausible.

One possibility is that ketamine exerts a direct effect on processes relevant to FND symptom generation. Several reports described rapid improvement in functional symptoms occurring shortly after ketamine administration, including acute functional motor paralysis and paradoxical vocal cord motion, raising the possibility that ketamine may transiently alter neural processes relevant to symptom expression. However, current evidence provides little support for a specific mechanistic pathway. Improvements were reported across highly heterogeneous symptom presentations and treatment contexts, and no study directly examined changes in FND-specific neurocognitive or neurophysiological processes. Consequently, any direct effect on functional symptom generation remains speculative.

An alternative explanation is that improvements in functional symptoms were secondary to broader psychological recovery rather than a direct effect on FND itself. Many contemporary reports involved patients receiving (Es)ketamine for TRD or other psychiatric conditions, and reductions in functional symptoms frequently occurred alongside improvements in depressive, anxiety, trauma-related, or general distress symptoms. Given the high prevalence of psychiatric comorbidity in FND (32), and the established associations between emotional distress, disability, coping difficulties, and symptom severity (33), ketamine may reduce factors that perpetuate or amplify symptom expression without directly targeting the underlying mechanisms of FND. Reported functional improvements may therefore have occurred indirectly through reductions in depression, anxiety, trauma-related symptoms, psychological distress, or cognitive rigidity (34). Emerging observational literature has also suggested that esketamine treatment may be associated with changes in domains such as cognitive rigidity, mentalization, social cognition, and psychological flexibility (35, 36), although the relevance of these findings to FND remains uncertain.

A related possibility is that ketamine functions primarily as a facilitator of psychological change. Historically, ketamine was administered within narcoanalytic and abreactive frameworks intended to enhance emotional processing, recollection of stressful experiences, and therapeutic engagement. Similarly, several contemporary reports incorporated (Es)ketamine-assisted psychotherapy, integration sessions, supportive psychotherapy, or ongoing psychological treatment. In such contexts, ketamine may facilitate emotional processing, enhance cognitive and psychological flexibility, promote insight, and increase engagement with therapy. Consequently, clinical improvement may arise through an interaction between ketamine-induced neurobiological effects and concurrent psychotherapeutic processes, as has been proposed in TRD and other conditions (37–39). This interpretation may be particularly relevant in cases where symptom improvement followed emotionally salient experiences or occurred in conjunction with structured psychotherapeutic interventions.

The diversity of treatment contexts across the literature makes these mechanisms difficult to disentangle. While some reports administered (Es)ketamine specifically to target functional symptoms, many involved treatment of comorbid psychiatric disorders. Consequently, reported improvements may reflect direct effects on FND, indirect benefits arising from psychiatric recovery, facilitation of psychotherapeutic processes, or broader non-specific therapeutic factors. The currently available literature does not allow these competing explanations to be distinguished.

The treatment context itself may also contribute substantially to observed improvement. Most included reports involved highly intensive clinical environments characterised by specialist attention, careful monitoring, psychoeducation, therapeutic engagement, and strong treatment expectations. Such factors are increasingly recognised as important contributors to clinical outcomes in FND, where diagnostic explanation, therapeutic alliance, patient expectations, and engagement in treatment can independently influence symptom improvement (38, 40, 41). Many interventions were delivered as novel or highly specialised treatments, circumstances known to enhance expectancy effects. This explanation is especially relevant given the reliance on uncontrolled case reports, subjective outcome measures, and clinician-observed improvement. Functional symptoms are known to be sensitive to attention, expectation, symptom beliefs, and contextual influences. Consequently, part or all of the reported benefit may reflect non-specific therapeutic effects rather than a unique property of ketamine itself (38).

Finally, spontaneous improvement cannot be excluded. Many functional symptoms fluctuate over time, and some presentations improve rapidly following diagnostic clarification, changes in clinical context, reassurance, removal of stressors, or resolution of acute physiological or psychological triggers (42, 43). Acute presentations may be particularly susceptible to regression toward the mean, whereby symptoms naturally improve following periods of peak severity (44). This possibility is especially relevant for emergency department reports describing rapid symptom resolution following a single ketamine administration. Without comparator groups, it is impossible to determine whether similar improvement would have occurred in the absence of ketamine treatment.

The available literature is insufficient to discriminate between these explanatory models. Indeed, multiple mechanisms may operate simultaneously within individual patients. Future studies should therefore move beyond simple symptom outcomes and incorporate measures capable of distinguishing direct effects on functional symptoms from improvements mediated through psychiatric recovery, psychotherapeutic engagement, expectancy effects, or natural symptom variability. Until such data become available, mechanistic interpretations should remain cautious.

### Dissociative phenomena with (Es)ketamine

Dissociative phenomena may be particularly relevant when considering (Es)ketamine-based interventions in FND. Contemporary models recognise that dissociative symptoms occur in a subset of individuals with FND and may involve disturbances in agency, embodiment, attention, memory, and bodily awareness (2, 45–47). Because (Es)ketamine reliably induces transient alterations in perception, self-experience, and embodiment, it has been hypothesised that these experiences could influence functional symptoms by increasing cognitive or behavioural flexibility, modifying self-processing, or facilitating engagement with psychological interventions (35, 48–50). However, the available evidence provides little direct support for or against this hypothesis.

Recent qualitative and phenomenological studies suggest that ketamine-induced dissociative experiences often differ from pathological dissociation and are highly heterogeneous, encompassing alterations in temporal perception, bodily and spatial awareness, sensory processing, and subjective distance from emotional distress (35, 48, 51). These experiences may be perceived as neutral, meaningful, or occasionally distressing, with their interpretation influenced by factors such as treatment expectations, therapeutic framing, preparation, monitoring, and post-session integration (51). Despite growing interest in these phenomena, current evidence does not establish whether dissociation contributes to therapeutic benefit, is clinically neutral, or may be destabilising in susceptible individuals (12, 52).

Notably, none of the studies included in this review systematically assessed dissociative experiences or examined their relationship with treatment response. Consequently, it remains unclear whether dissociative phenomena contribute to clinical improvement, represent an epiphenomenon of broader neurobiological processes, or are unrelated to treatment outcomes. Although dissociative experiences have historically been proposed as a potential mechanism of change, emerging evidence from depression research suggests that therapeutic benefit can occur in the absence of prominent dissociation and that associations between dissociative intensity and treatment response are inconsistent (12, 51, 52).

This question may be particularly important in FND, where disturbances of agency, bodily ownership, embodiment, and self-experience are common in some patients. While transient alterations in these domains could theoretically influence symptom processing, they may not be uniformly beneficial and could be distressing for some individuals, particularly those with prominent dissociative symptoms, trauma-related psychopathology, or heightened sensitivity to altered states of consciousness (53). Future studies should therefore prospectively assess the nature, severity, and subjective meaning of dissociative experiences during treatment and determine whether they are associated with therapeutic benefit, treatment tolerability, or adverse outcomes.

### Adverse effects and tolerability

The available literature suggests that (Es)ketamine were generally well tolerated in the reported FND populations. Adverse effects were predominantly transient and mild, consisting mainly of dissociative or perceptual experiences, dizziness, nausea, headache, transient confusion, and modest cardiovascular changes. No study reported persistent psychosis, prolonged cognitive impairment, treatment discontinuation attributable to adverse effects, or serious treatment-related medical complications. These findings are broadly consistent with the wider ketamine literature, in which transient dissociative symptoms and short-lived increases in blood pressure represent the most commonly observed adverse effects in supervised clinical settings (54–56). However, the significance and tolerability of these experiences have not been systematically studied in FND populations. Although dissociative effects are generally described as transient and manageable, isolated case reports and qualitative studies suggest that some individuals may experience ketamine-induced dissociation as distressing, unsettling, or even traumatic (57).

Nevertheless, interpretation of the apparent safety profile is limited by the low quality of the evidence base, brief and non-standardised adverse-event reporting, and the potential for publication bias favouring favourable outcomes and good tolerability over negative experiences, symptom worsening, or treatment-emergent adverse effects.

### Limitations

Several limitations should be considered when interpreting these findings. First, the evidence base was extremely limited and consisted predominantly of uncontrolled observations. Consequently, this review cannot establish efficacy, estimate treatment effects, or determine whether reported improvements exceeded spontaneous recovery, symptom fluctuation, regression to the mean, expectancy effects, or other non-specific therapeutic factors. Second, substantial clinical and methodological heterogeneity was present across studies. Included reports differed in diagnostic terminology, symptom phenotype, (Es)ketamine formulation, route of administration, dose, treatment setting, follow-up duration, treatment intent, co-interventions, and outcome assessment. Historical diagnoses such as conversion reaction, dissociative reaction, and hysteria do not map directly onto contemporary FND classifications, while several studies involved FND-adjacent conditions.

Furthermore, ketamine was administered either to target functional symptoms directly or to treat psychiatric disorders such as TRD or PTSD, with functional improvement often reported as a secondary outcome. Concurrent psychotherapy, ketamine-assisted therapy, rehabilitation, medication changes, and ongoing psychiatric care were common. As a result, the independent contribution of ketamine to observed outcomes cannot be determined in most cases, and confidence in generalising findings to contemporary FND populations is limited.

Third, outcome assessment was generally weak. Only one study employed validated FND-specific outcome measures, with most relying on clinician impression, narrative symptom descriptions, patient self-report, seizure frequency, or psychiatric rating scales. This limits comparison across studies and prevents robust assessment of symptom severity, functional impairment, durability of response, and patient-centred recovery.

Fourth, the literature is vulnerable to publication and reporting bias. Positive or unusual outcomes are more likely to be published than treatment failures, adverse events, or neutral findings, potentially inflating the apparent therapeutic promise of (Es)ketamine. Adverse-event reporting was inconsistent and follow-up was often limited, preventing firm conclusions regarding safety.

Finally, several limitations relate to the review process itself. The review was not prospectively registered, and title/abstract screening and full-text assessment were performed by a single reviewer, creating potential selection bias despite efforts to minimise this risk. The search strategy relied primarily on Google Scholar and PubMed, with PsycINFO added retrospectively following peer review; Embase, Scopus/Web of Science, and Cochrane were not systematically searched. Google Scholar screening was limited to the first 1,000 relevance-ranked results for large searches, and only English-language peer-reviewed publications were included. Given the small number of studies, marked heterogeneity, and absence of controlled designs, quantitative synthesis was not possible. Accordingly, the findings should be regarded as descriptive and hypothesis-generating rather than evidence supporting routine clinical use of (Es)ketamine for FND.

### Future directions

Historical pharmacological interventions in FND, including abreaction, therapeutic propofol sedation, ECT, and more recently ketamine, have all followed a similar trajectory: encouraging case-level observations without subsequent rigorous evaluation (6, 8, 58). Similarly, psychedelic therapies were described in historical reports but subsequently fell out of clinical use because of limited evidence (59, 60). Psychedelic research illustrates that historical observations can be revisited using contemporary trial methodology (61, 62). A similar research pathway may now be appropriate for (Es)ketamine in FND. Rather than supporting routine clinical use, the current literature provides a rationale for carefully designed mechanistic and clinical studies to determine whether preliminary observations translate into meaningful therapeutic benefit. Future research should prioritise prospective studies specifically designed to evaluate (Es)ketamine in well-characterised FND populations using contemporary diagnostic criteria and validated FND-specific outcome measures.

A key priority is distinguishing studies in which (Es)ketamine is administered to target functional symptoms directly from those in which it is prescribed primarily for psychiatric comorbidity, such as TRD or PTSD. Careful documentation of concurrent interventions, including psychotherapy, rehabilitation, and medication changes, will be essential to reduce confounding and clarify treatment effects. Longer follow-up periods are also needed to determine the durability of any observed benefits. Recent clinical frameworks developed for esketamine treatment in TRD emphasise integrated models involving psychiatric supervision, nursing monitoring, psychological preparation, treatment-session support, and post-treatment integration (63). Although such models have not been evaluated in FND, they may provide a useful framework for future studies, particularly given the importance of psychoeducation, behavioural rehabilitation, psychological treatment, and symptom meaning within contemporary FND care. Similar paradigm has been utilised in FND studies using psilocybin (36, 61, 64).

The role of dissociative experiences warrants particular attention. Future studies should prospectively assess dissociative phenomena and examine whether they influence treatment response, tolerability, or adverse outcomes. Potential mediators such as cognitive flexibility, mentalization, altered interoception and psychological engagement should also be explored, although their relevance to FND remains uncertain (35, 65).

A pragmatic next step would be an open-label feasibility study of intranasal esketamine within specialist multidisciplinary FND services. Primary outcomes should focus on feasibility, acceptability, safety, and retention, with secondary assessment of functional symptoms, disability, quality of life, psychiatric symptoms, and healthcare utilisation. Only if such studies demonstrate acceptable safety and a signal of potential benefit should adequately powered controlled trials be undertaken. Ultimately, the field requires studies capable of determining whether ketamine-based interventions confer benefits beyond those attributable to psychiatric recovery, concurrent therapies, expectancy effects, and the natural variability of functional symptoms.

### Conclusion

Published evidence for (Es)ketamine in FND is limited to uncontrolled, diagnostically heterogeneous reports with substantial confounding by psychiatric comorbidity and concurrent interventions. Existing data are insufficient to support clinical efficacy or routine use. The main value of the literature is hypothesis generation and justification for carefully designed feasibility studies using contemporary FND criteria, validated outcomes, prospective adverse-event monitoring, and clear separation of functional and psychiatric endpoints.

## Data Availability

The original contributions presented in the study are included in the article/**Supplementary Material**. Further inquiries can be directed to the corresponding author.

## Supplementary material

Supplementary Table 1 Search strategy and number of records retrieved. Searches were conducted from database inception to 31 December 2025 using PubMed, Google Scholar, and a retrospective PsycINFO search. Free-text search terms were used because of the historical evolution and heterogeneity of FND terminology. MeSH terms were not used, as many historical reports and case studies relevant to contemporary FND terminology are inconsistently indexed. Each exposure term (“ketamine”, “esketamine”, or “NMDA antagonist”) was paired with each functional term using the Boolean operator “AND”. Google Scholar searches yielding more than 1,000 results were limited to the first 1,000 relevance-ranked records screened. The PsycINFO search excluded PubMed-indexed articles.** PsycINFO was searched retrospectively following peer-review suggestion using identical search terms while excluding PubMed-indexed records. † Google Scholar searches returning more than 1,000 results were limited to screening the first 1,000 records sorted by relevance. Search dates: Google Scholar and PubMed searches were performed in January 2026 and included records indexed from database inception to 31 December 2025. PsycINFO was searched retrospectively on 14 June 2026 using the same paired search terms.*
