# Supplementary material for: (Es)ketamine in functional neurological disorder: a systematic review
Source: Front Psychiatry. 2026 Jul 20;17:1872389. doi: 10.3389/fpsyt.2026.1872389 (PMC13430003; doi:10.3389/fpsyt.2026.1872389)
Supplement: Supplementary Table 1 — Search strategy and number of records retrieved. Searches were conducted from database inception to 31 December 2025 using PubMed, Google Scholar, and a retrospective PsycINFO search. Free-text search terms were used because of the historical evolution and heterogeneity of FND terminology. MeSH terms were not used, as many historical reports and case studies relevant to contemporary FND terminology are inconsistently indexed. Each exposure term (“ketamine”, “esketamine”, or “NMDA antagonist”) was paired with each functional term using the Boolean operator “AND”. Google Scholar searches yielding more than 1,000 results were limited to the first 1,000 relevance-ranked records screened. The PsycINFO search excluded PubMed-indexed articles.* PsycINFO was searched retrospectively following peer-review suggestion using identical search terms while excluding PubMed-indexed records. † Google Scholar searches returning more than 1,000 results were limited to screening the first 1,000 records sorted by relevance. Search dates: Google Scholar and PubMed searches were performed in January 2026 and included records indexed from database inception to 31 December 2025. PsycINFO was searched retrospectively on 14 June 2026 using the same paired search terms. [file SupplementaryFile1.docx]

Tamilson B, Chitolie M, Coebergh J, Agrawal N, Poole N, Kanaan R and Nicholson T, Butler M (2026) (Es)ketamine in functional neurological disorder: a

systematic review. Front. Psychiatry 17:1872389. doi:

**Supplementary Table S1. Search Strategy and Number of Records Retrieved**

Searches were conducted from database inception to 31 December 2025 using PubMed, Google Scholar, and a retrospective PsycINFO search. Free-text search terms were used because of the historical evolution and heterogeneity of FND terminology. MeSH terms were not used, as many historical reports and case studies relevant to contemporary FND terminology are inconsistently indexed. Each exposure term ("ketamine", "esketamine", or "NMDA antagonist") was paired with each functional term using the Boolean operator “AND”. Google Scholar searches yielding more than 1,000 results were limited to the first 1,000 relevance-ranked records screened. The PsycINFO search excluded PubMed-indexed articles.

| Exposure term | Functional term | Google Scholar hits | PubMed hits | PsycINFO hits* |
| --- | --- | --- | --- | --- |
| “Ketamine” | “Functional neurological disorder” | 190 | 1 | 0 |
| “Ketamine” | “Conversion disorder” | 549 | 2 | 0 |
| “Ketamine” | “Functional symptoms” | 247 | 0 | 1 |
| “Ketamine” | “Psychogenic” | 192 | 6 | 1 |
| “Ketamine” | “Non-epileptic seizures” | 347 | 1 | 0 |
| “Ketamine” | “PNES” | 197 | 0 | 0 |
| “Ketamine” | “Functional movement disorder” | 33 | 0 | 0 |
| “Ketamine” | “Dissociative” | 1,000† | 443 | 5 |
| “Esketamine” | “Functional neurological disorder” | 68 | 1 | 1 |
| “Esketamine” | “Conversion disorder” | 49 | 0 | 0 |
| “Esketamine” | “Functional symptoms” | 28 | 0 | 0 |
| “Esketamine” | “Psychogenic” | 153 | 0 | 1 |
| “Esketamine” | “Non-epileptic seizures” | 23 | 0 | 0 |
| “Esketamine” | “PNES” | 18 | 0 | 0 |
| “Esketamine” | “Functional movement disorder” | 9 | 0 | 0 |
| “Esketamine” | “Dissociative” | 1,000† | 40 | 68 |
| “NMDA antagonist” | “Functional neurological disorder” | 9 | 0 | 0 |
| “NMDA antagonist” | “Conversion disorder” | 82 | 0 | 0 |
| “NMDA antagonist” | “Functional symptoms” | 58 | 0 | 1 |
| “NMDA antagonist” | “Psychogenic” | 615 | 0 | 0 |
| “NMDA antagonist” | “Non-epileptic seizures” | 76 | 0 | 0 |
| “NMDA antagonist” | “PNES” | 17 | 0 | 0 |
| “NMDA antagonist” | “Functional movement disorder” | 2 | 0 | 0 |
| “NMDA antagonist” | “Dissociative” | 1,000† | 7 | 2 |
| Total retrievals | | **5,962** | **501** | **80** |

** PsycINFO was searched retrospectively following peer-review suggestion using identical search terms while excluding PubMed-indexed records.*

*† Google Scholar searches returning more than 1,000 results were limited to screening the first 1,000 records sorted by relevance.*

*Search dates: Google Scholar and PubMed searches were performed in January 2026 and included records indexed from database inception to 31 December 2025. PsycINFO was searched retrospectively on 14 June 2026 using the same paired search terms.*
